# Cyclin I-like (CCNI2) is a cyclin-dependent kinase 5 (CDK5) activator and is involved in cell cycle regulation

**DOI:** 10.1038/srep40979

**Published:** 2017-01-23

**Authors:** Chengcheng Liu, Xiaoyan Zhai, Bin Zhao, Yanfei Wang, Zhigang Xu

**Affiliations:** 1 Shandong Provincial Key Laboratory of Animal Cells and Developmental Biology, Shandong University School of Life Sciences, Jinan, Shandong 250100, China

## Abstract

In contrast to conventional cyclin-dependent kinases that are important for mitotic cell division, cyclin-dependent kinase 5 (CDK5) is predominantly activated in post-mitotic cells and is involved in various cellular events. The kinase activity of CDK5 is tightly regulated by specific activators including p35, p39, and cyclin I (CCNI). Here we show that cyclin I-like (CCNI2), a homolog of CCNI, interacts with CDK5 and activates the kinase activity of CDK5. Different from CCNI, which colocalizes with CDK5 in the nuclei in transfected cells, CCNI2 mainly retains CDK5 in the cytoplasm as well as on the cell membrane. Furthermore, although the expression level of *CCNI2* mRNA and CCNI2 protein do not change significantly during cell cycle, depletion of CCNI2 with siRNA affects cell cycle progression as well as cell proliferation. In conclusion, our data strongly suggest that CCNI2 is a novel CDK5 activator and is involved in cell cycle regulation.

Cyclin-dependent kinase 5 (CDK5) is a proline-directed serine/threonine kinase. Despite the high degree of homology between CDK5 and other conventional cyclin-dependent kinases (CDKs) that are important for mitotic cell division, CDK5 usually is not directly involved in cell cycle regulation. Instead, it is abundantly expressed in post-mitotic cells such as neurons and is necessary for neural differentiation^1^,^2^,^3^. It is now well accepted that CDK5 plays important roles in multiple cellular events, such as cytoskeletal dynamics, signaling cascades, gene expression, and cell survival^4^,^5^. As a result, CDK5 dysregulation has been implicated in various diseases, such as Alzheimer’s disease (AD), amyotrophic lateral sclerosis (ALS), and Parkinson’s disease (PD)^4^,^5^.

Similar to conventional CDKs, whose kinase activity needs to be activated by cyclins, CDK5 needs to be activated by specific activators. The first characterized CDK5 activator is p35, which is predominantly expressed in neurons^6^,^7^. Although the primary sequence of p35 is distinct from that of cyclins, it adopts a tertiary structure similar to that of cyclins^8^. Notably, p35 consists of two parts, an N-terminal p10 fragment and a C-terminal p25 fragment. Under neurotoxic stress, p35 is cleaved into p25 and p10 by calpain^9^,^10^. Because p10 contains a signal for degradation through ubiquitin-proteasome pathway, after cleavage, p25 is resistant to ubiquitin-mediated proteolysis and has a much longer half-life than p35. As a result, p25 constitutively activates CDK5 and promotes neurodegeneration^9^,^10^. The second characterized CDK5 activator is p39, a homolog of p35 that is also predominantly expressed in neurons^11^. Similar to p35, p39 could be cleaved into p10 and p29, which contributes to deregulation of CDK5^12^. Although p35 or p39 single knockout mice are viable and only show mild phenotypes, p35 and p39 double-knockout mice display perinatal lethality with extensive defects in brain development and neuronal differentiation^13^,^14^,^15^. These phenotypes are identical to those of Cdk5 knockout mice^16^, suggesting that p35 and p39 are the main Cdk5 activators in the brain.

Recently, cyclin I (CCNI) was identified as a new CDK5 activator^17^. Different from p35 and p39, CCNI contains a typical cyclin box^18^, which is responsible for binding and activating CDKs^19^,^20^. Based on sequence similarity, CCNI, cyclin G1 (CCNG1), and cyclin G2 (CCNG2) form a distinct sub-group in the cyclin family^21^. CCNI is broadly expressed in various tissues, and is involved in apoptosis and cell cycle regulation^22^,^23^. Ccni knockout mice are viable and do not have any apparent developmental defects. However, following induction of experimental glomerulonephritis, Ccni knockout mice showed dramatically decreased renal function, accompanied with increased podocyte apoptosis^22^.

In this study, we show that cyclin I-like (CCNI2) is a novel CDK5 activator. CCNI2 is a homolog of CCNI, and at present its function is largely unknown. We confirmed that CCNI2 binds CDK5 and activates CDK5 kinase activity. Different from CCNI, which colocalizes with CDK5 in the nuclei in cultured cells, CCNI2 mainly retains CDK5 in the cytoplasm as well as on the cell membrane. Furthermore, depletion of CCNI2 with siRNA inhibits cell cycle progression and cell proliferation.

## Results

### CCNI2 is a novel CDK5-binding partner

**Figure 1:**
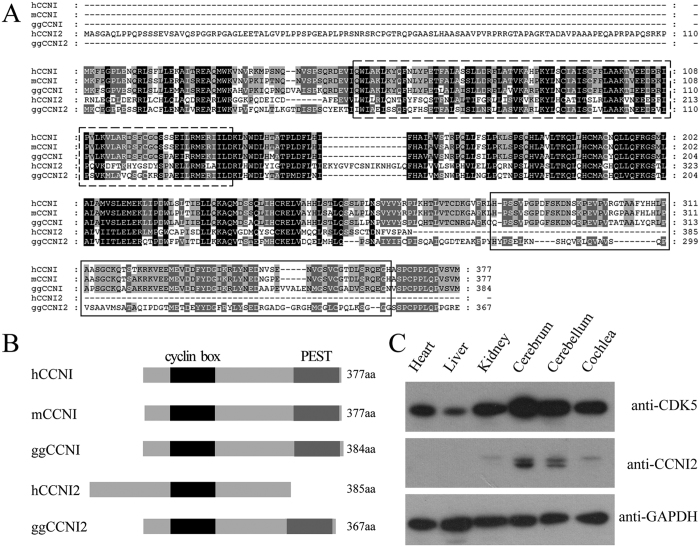
Protein sequence and tissue expression pattern of CCNI2. (**A**) Amino acid sequence alignment of CCNI and CCNI2 from different species. Amino acid sequences of *Homo sapiens* CCNI, *Mus musculus* CCNI, *Gallus gallus* CCNI and *Homo sapiens* CCNI2, *Gallus gallus* CCNI2 were aligned using the ClustalW method. Dashed box indicates the cyclin box. Solid box indicates the PEST region. (**B**) Schematic representation of the domain structures of CCNI and CCNI2. Cyclin box and PEST region are indicated. (**C**) Tissue expression pattern of chicken CCNI2 was examined by western blot. Total proteins from E18.5 chicken tissues were extracted and separated by PAGE and detected with antibodies against CDK5, CCNI2, and GAPDH.

In an effort to identify new CDK5 binding-proteins, we performed yeast two-hybrid screening of a chicken cochlear cDNA library using CDK5 as bait. The identified positive clones encode two proteins, cyclin I (CCNI, GenBank accession number XP_420590) and cyclin I-like (CCNI2, GenBank accession number XP_001234830) (Table 1). CCNI was originally identified based on its similarity to other known cyclins^18^, and has been shown to bind and activate CDK5^17^. CCNI2 is considered as a homolog of CCNI, although the overall similarity between these two proteins is relatively low except for the cyclin box (Fig. 1A,B). At present, the physiological function of CCNI2 is largely unknown, and the interaction between CCNI2 and CDKs has not been reported.

Sequence analysis revealed that CCNI2 is not conserved during evolution. *CCNI2* gene is present in human, chicken and zebrafish genomes, but not in mouse and rat genomes. The expression pattern of CCNI2 protein in different chicken tissues was examined by western blot with a polyclonal CCNI2 antibody. The results showed that chicken CCNI2 is highly expressed in neural tissues including cerebrum and cerebellum, and weakly expressed in kidney and cochlea, whereas undetected in liver and heart (Fig. 1C).

**Figure 2:**
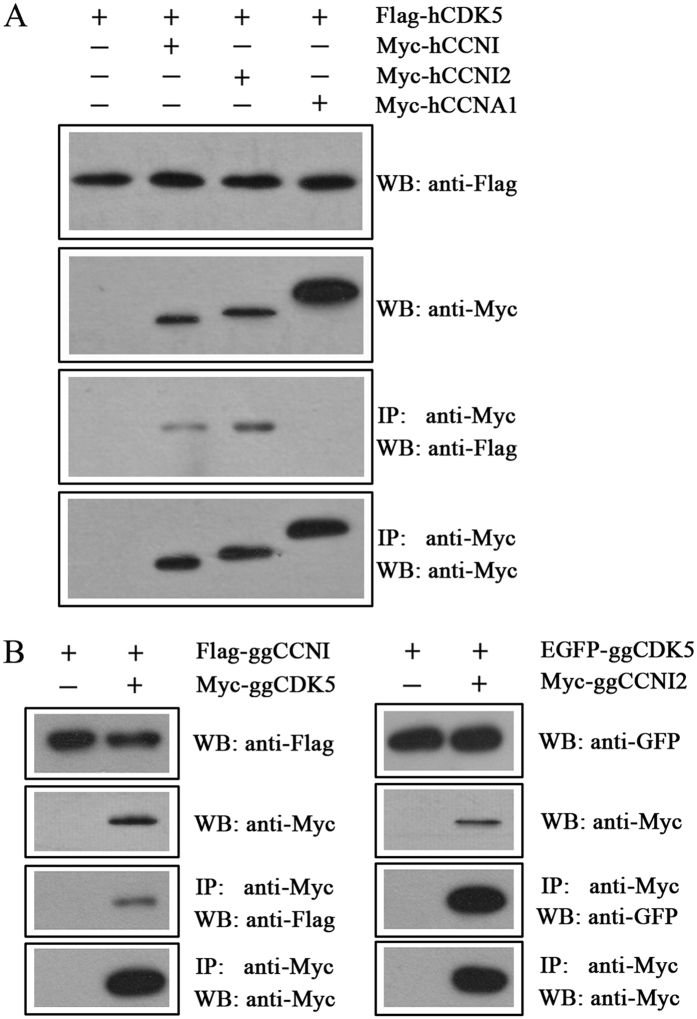
CCNI2 interacts with CDK5. Western blots show that CCNI and CCNI2 were co-immunoprecipitated with CDK5. Expression vectors were transfected into HEK293 cells to express epitope-tagged human (**A**) or chicken (**B**) CDK5, CCNI, and CCNI2 proteins, and cell lysates were subjected to immunoprecipitation. Human CCNA1 was included as a negative control. IP indicates antibody used for immunoprecipitation, and WB indicates antibody used for detection.

We then performed co-immunoprecipitation (co-IP) experiment to confirm the interaction between CCNI2 and CDK5. CCNI was also included in this experiment for comparison. The results revealed that Flag-tagged human CDK5 is co-immunoprecipitated with Myc-tagged human CCNI or CCNI2, but not cyclin A1 (CCNA1) (Fig. 2A). Noticeably, our results indicated that the interaction between CDK5 and CCNI2 is stronger than that between CDK5 and CCNI. The interaction was also confirmed by Co-IP when chicken CDK5, CCNI, and CCNI2 were used (Fig. 2B).

### CCNI2 colocalizes with CDK5 in cultured cells

**Figure 3:**
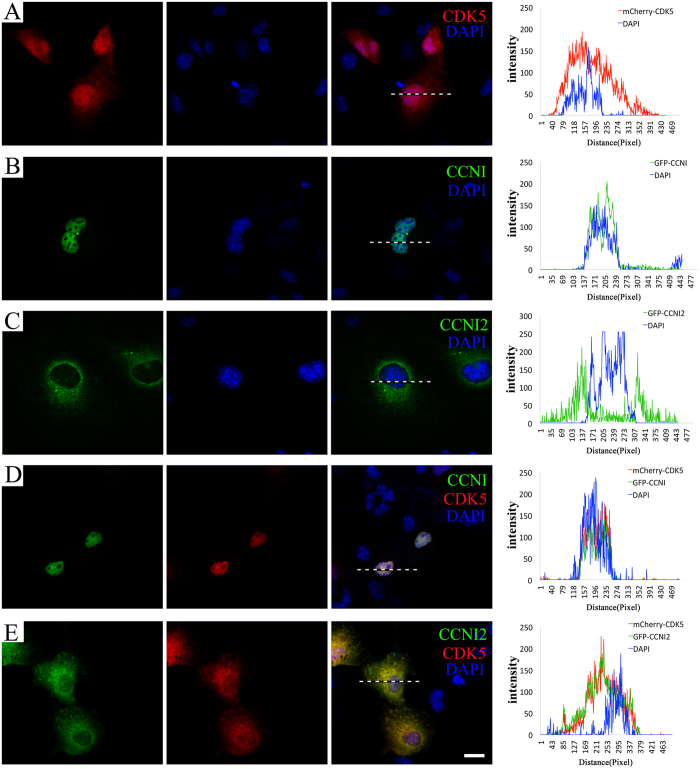
Colocalization of CCNI2 and CDK5 in transiently transfected cells. COS-7 cells were transfected with expression vectors that express human CCNI, CCNI2 and CDK5 with GFP or mCherry tag. (**A**) When expressed alone, mCherry-CDK5 localizes diffusely in the cell. (**B**) GFP-CCNI mainly localizes in the nuclei. (**C**) GFP-CCNI2 mainly localizes in the cytoplasm and the plasma membrane. (**D**) When expressed together with GFP-CCNI, mCherry-CDK5 moves into the nuclei and colocalizes with CCNI. (**E**) When expressed together with GFP-CCNI2, mCherry-CDK5 mainly localizes in the cytoplasm as well as on the plasma membrane. Nuclei were stained with DAPI. The fluorescent intensity was quantified using Image J. Scale bar: 20 μm.

We then examined the subcellular localization of CDK5 and CCNI2 in cultured cells. When overexpressed in COS-7 cells, mCherry-tagged human CDK5 is detected diffusely in the cell (Fig. 3A). In contrast, CCNI and CCNI2 have more specific subcellular localizations. GFP-tagged human CCNI mainly localizes in the nuclei, whereas CCNI2 mainly localizes in the cytoplasm and the plasma membrane (Fig. 3B,C). When expressed together with human CCNI, CDK5 moves into the nuclei and colocalizes with CCNI (Fig. 3D). In contrast, human CCNI2 mainly retains CDK5 in the cytoplasm as well as on the plasma membrane (Fig. 3E). Similar results were observed in HeLa cells and HEK293 cells (data not shown). Taken together, the fact that CCNI2 colocalizes with CDK5 in various cultured cells is consistent with our hypothesis that CCNI2 is a CDK5-binding partner.

### CCNI2 activates CDK5’s kinase activity

**Figure 4:**
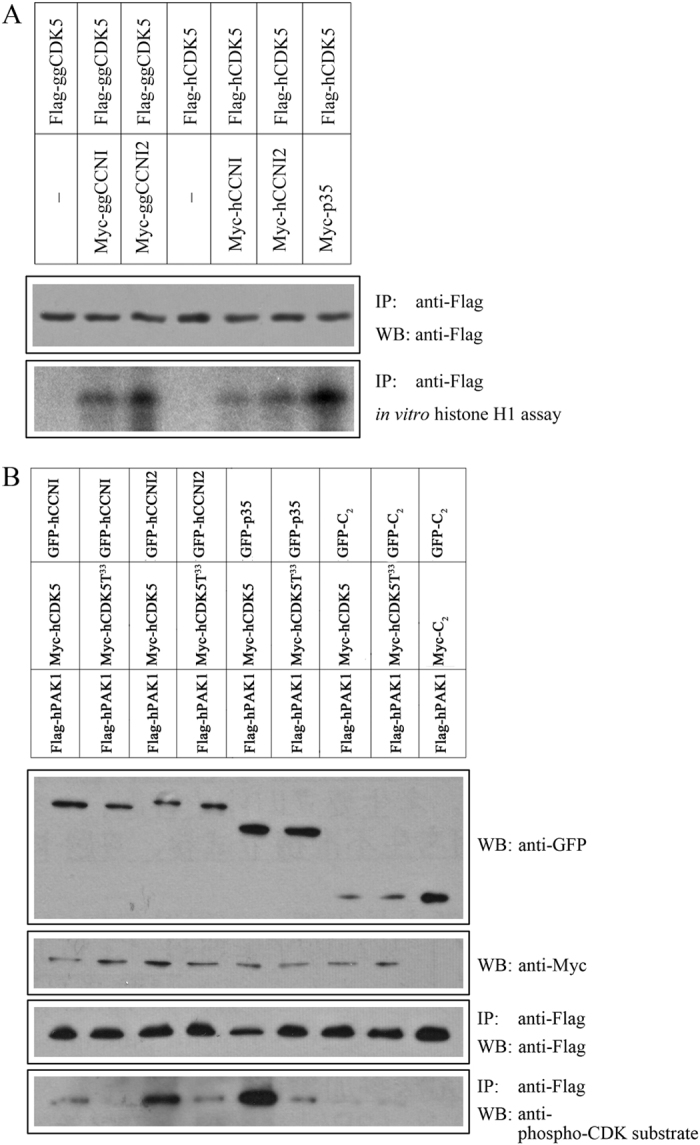
CCNI2 activates the kinase activity of CDK5. (**A**) *In vitro* histone H1 kinase assay was performed to examine the ability of human or chicken CCNI2 to activate CDK5. HEK293 cells were transfected with Myc-tagged CCNI/CCNI2/p35 and Flag-tagged CDK5, and immunoprecipitations were performed with anti-Flag agarose followed by histone H1 kinase assay. (**B**) PAK1 phosphorylation was used to examine the ability of human CCNI2 to activate CDK5. HEK293 cells were transfected with GFP-tagged CCNI/CCNI2/p35, Myc-tagged CDK5, and Flag-tagged PAK1, and immunoprecipitations were performed with anti-Flag agarose. Phosphorylation of immunoprecipitated PAK1 was examined with anti-phospho-CDK5 substrate antibody. Dominant-negative mutant CDK5 (CDK5T33) was included as a negative control.

To examine the possible regulation of CDK5 activity by CCNI2, HEK293 cells were transfected with plasmids expressing Flag-tagged CDK5 and Myc-tagged CCNI2. Myc-tagged CCNI and p35 were included as controls. Protein lysates of transfected cells were immunoprecipitated with an anti-Flag antibody, and histone H1 was used as kinase substrate to determine the kinase activity of these complexes. The result showed that CCNI2-CDK5 complex was able to phosphorylate histone H1, and this activity is stronger than that of CCNI-CDK5 complex, but weaker than that of p35-CDK5 complex (Fig. 4A). This is true for both human and chicken proteins, and is consistent with the finding that CCNI2 has a higher binding capacity with CDK5 when compared with CCNI (Fig. 2A).

We also examined the kinase activity of CDK5/CCNI2 using another known CDK5 substrate, PAK1^24^,^25^. HEK293 cells were transfected with plasmids expressing Flag-tagged human PAK1, Myc-tagged human CDK5, and GFP-tagged human CCNI, CCNI2 or p35. Flag-PAK1 was immunoprecipitated with anti-Flag antibody, and its phosphorylation status was examined by western blot using a phospho-CDK5 substrate antibody. The result showed that PAK1 phosphorylation is enhanced by the CDK5 complexes, and CDK5/CCNI2 activity is stronger than CDK5/CCNI, but weaker than CDK5/p35 (Fig. 4B), which is consistent with the histone H1 kinase assay result. The Lysine at codon 33 (K33) is important for the kinase activity of CDK5, and when this amino acid is changed to Threonine, the mutant CDK5 (CDK5T33) acts as a dominant-negative mutant^3^. When wild-type CDK5 was replaced with CDK5T33, phosphorylation of PAK1 was largely abolished (Fig. 4B), confirming that in our experiment PAK1 phosphorylation is indeed mediated by CDK5.

### CCNI2 depletion affects cell cycle progression and cell proliferation

**Figure 5:**
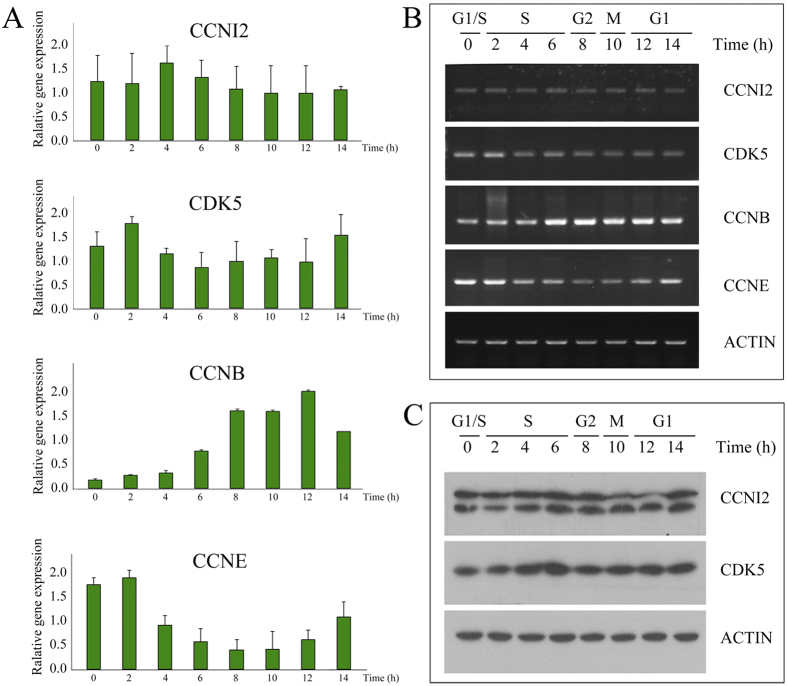
Expression analysis of CCNI2 during cell cycle. HeLa cells were arrested at late G_1_ phase with double-thymidine block, then released to enter S phase. Total RNA from different time point was extracted and used as template for reverse transcription. Quantitative PCR (**A**) and semiquantitative PCR (**B**) were performed using this cDNA as template. *CCNE* and *CCNB* were used as cell cycle markers of G_1_/S and G_2_/M phases, respectively. (**C**) Lysates from cells at different time points were subjected to SDS-PAGE, followed by western blot with different antibodies as indicated.

To explore whether CCNI2 plays a role in cell cycle regulation, we first examined the expression level of *CCNI2* mRNA and CCNI2 protein during cell cycle. HeLa cells were synchronized at late G1 phase with a double-thymidine block, then released into cell cycle and collected at various time points. The cells harvested at different time points were classified into different cell cycle phases according to *cyclin E (CCNE*) and *cyclin B (CCNB*) mRNAs expression level, which act as G1/S-phase marker and G2/M-phase marker, respectively (Fig. 5A,B). The results showed that *CCNI2* mRNA level largely remains constant during cell cycle (Fig. 5A,B). We also performed western blot using a CCNI2-specific antibody, which showed that CCNI2 protein level also does not change significantly during cell cycle (Fig. 5C).

**Figure 6:**
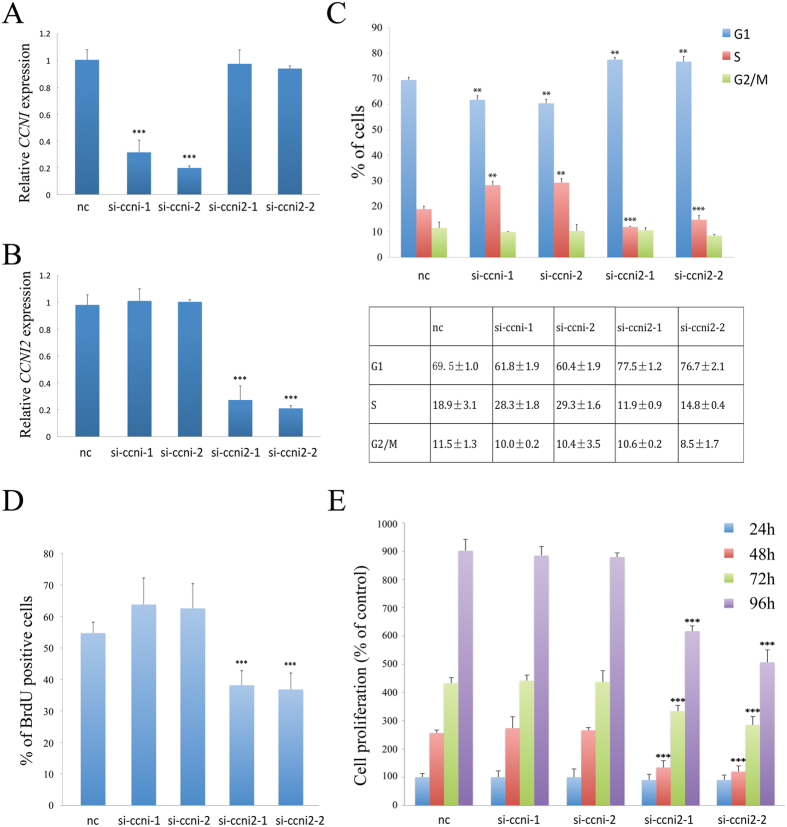
Knockdown of CCNI2 decreases cell cycle progression and cell proliferation of HeLa cells. The specificity and efficiency of siRNAs against CCNI (**A**) and CCNI2 (**B**) were examined by quantitative PCR. HeLa cells were transfected with siRNAs at day 0 and day 1, and the expression level of *CCNI* or *CCNI2* was determined by qPCR analysis at day 2. (**C**) Cell cycle profiles of CCNI-depleted and CCNI2-depleted HeLa cells. (**D**) BrdU incorporation of CCNI-depleted and CCNI2-depleted HeLa cells. (**E**) Cell proliferation of CCNI-depleted and CCNI2-depleted HeLa cells was examined by MTT assay. The bar graphs and the table show quantification of the results, with each value represents the mean ± SD of three independent experiments. Statistical significance is shown using the Student *t* test analysis; *P < 0.05; **P < 0.01; ***P < 0.001.

We next analyzed the effect of CCNI2 depletion with siRNA on cell cycle progression in HeLa cells. CCNI has been shown to regulate cell cycle^23^, hence we also examined the effect of CCNI depletion with siRNA in our system. HeLa cells were transfected with siRNA, and the efficiency of siRNA knockdown was evaluated by quantitative real time PCR and western blot (Fig. 6A,B, Fig. S1). The effect of siRNA on cell cycle progression was then determined by flow cytometry. When CCNI2 was depleted, the number of cells at G1 phase increases, concomitant with a decrease in the number of cells at S phase. In contrast, in CCNI-depleted cells, the number of cells at G1 phase decreases, concomitant with an increase in the number of cells at S phase (Fig. 6C). Similar result was obtained when A549 cells were used (Fig. S2A). Furthermore, we found that this effect is CDK5-dependent, since CCNI or CCNI2 depletion no longer affects cell cycle progression when CDK5 is also depleted (Fig. S3).

We then performed bromodeoxyuridine (BrdU) incorporation experiment to confirm the effect of CCNI2 depletion on cell cycle regulation. BrdU incorporation of siRNA-transfected HeLa cells was determined by immunostaining with an anti-BrdU antibody. Consistent with the result of flow cytometry, BrdU incorporation is decreased in CCNI2-depleted cells. In CCNI-depleted cells, BrdU incorporation is slightly increased, although the increase is not statistically significant (Fig. 6D). At last, proliferation of siRNA-transfected HeLa cells was examined by performing MTT assay. The result showed that CCNI2 depletion effectively decreases cell proliferation, whereas CCNI depletion has no effect (Fig. 6E). Similar result was obtained when A549 cells were used (Fig. S2B,C).

## Discussion

### CCNI2 is a novel CDK5 activator

As an important kinase, CDK5 requires other proteins to activate its kinase activity. Several CDK5 activators have been identified, including p35, p39, and CCNI^6^,^7^,^11^,^17^. Here we demonstrate that CCNI2 functions as a novel CDK5 activator. CCNI2 physically interacts with CDK5 and activates its kinase activity. Moreover, compared to the previously identified CDK5 activator CCNI, CCNI2 exhibits higher binding affinity with CDK5 and is more potent to activate CDK5.

Different from other known CDK5 activators, CCNI2 is not conserved during evolution. *CCNI2* gene and CCNI2 protein are missing in many species, including mouse and rat. Although CCNI2 is present in human and chicken, the N-terminus and C-terminus differ significantly among these species. Human CCNI2 has a unique long N-terminal fragment, which is not present in chicken CCNI2 and in CCNI. In addition, unlike chicken CCNI2 and CCNI, human CCNI2 lacks the C-terminal PEST region. Nevertheless, both human and chicken CCNI2 share a conserved cyclin box, which is responsible for binding and activating CDKs. Indeed, our data clearly show that both human and chicken CCNI2 bind and activate CDK5.

### CCNI2 and CCNI differently regulate the subcellular localization of CDK5

It has been shown that the physiological function of CDK5 depends on its subcellular localization^26^,^27^,^28^. Meanwhile, several reports suggest that the subcellular localization of CDK5 is determined by the subcellular localization of its activators. For example, p35 and p39 mediate the cytoplasm and membrane localization of CDK5^29^,^30^. Calpain-mediated cleavage of p35 to p25, in contrast, leads to nuclear accumulation of CDK5 and degenerative phenotypes^9^,^31^.

Our results suggest that CCNI2 also could regulate the subcellular localization of CDK5. When overexpressed alone in cultured cells, human CCNI2 localizes in the cytoplasm and the plasma membrane, whereas CDK5 localizes diffusely in the cell. When overexpressed together, however, CCNI2 mainly retains CDK5 in the cytoplasm as well as on the plasma membrane. This activity is very different from that of CCNI, which retains CDK5 in the nuclei. Recently, the subcellular localization of CDK5 was examined in p35 and Ccni knockout mice. The results revealed that p35 and CCNI are necessary for retaining CDK5 in the cytoplasm/plasma membrane or in the nuclei, respectively^32^. Our present data suggest that CCNI2 and p35 target CDK5 to a similar subcellular distribution. p35 is predominantly expressed in neuronal tissues, whereas CCNI2 has a relatively more ubiquitous expression profile. Therefore CCNI2 might execute similar functions in determining the subcellular localization of CDK5 in non-neuronal tissues as p35 does in neurons.

The localization of p35 and p39 on the plasma membrane depends on their N-terminal myristoylation motif^9^,^29^. In addition, phosphorylation of p35 and p39 by CDK5 also affects their subcellular localization^30^. Sequence analysis of CCNI2 does not reveal the existence of a myristoylation motif. Nevertheless, GPS 2.1 analysis^33^ predicts two highly possible CDK5 phosphorylation sites in CCNI2, Ser21 and Ser42. Western blot with a specific anti-CCNI2 antibody revealed two bands from tissues and cultured cells, which might be caused by phosphorylation (Figs 1C and 5C). Further investigation is needed to examine the possible posttranslational modifications of CCNI2 and their biological significance.

### CCNI2 regulates cell cycle

In contrast to conventional CDKs, CDK5 is abundantly expressed in post-mitotic cells such as neurons^1^,^2^,^3^, and it was believed that CDK5 is not directly involved in cell cycle regulation. Recently, the role of CDK5 in cell cycle regulation has been reconsidered. Several groups showed independently that CDK5 could regulate cell cycle^26^,^27^,^34^,^35^,^36^,^37^. CCNI might play an important role in cell cycle regulation through CDK5. Although *CCNI* mRNA remains constant throughout different cell cycle phases^18^,^38^, CCNI protein expression level oscillates during cell cycle in synchronized HeLa cells, and CCNI knockdown causes G2/M arrest and inhibits cell cycle progression^23^.

In the present study we show that CCNI2 is also involved in cell cycle regulation. Although neither *CCNI2* mRNA nor CCNI2 protein level changes significantly during cell cycle, CCNI2 depletion with siRNA decreases cell cycle progression. Furthermore, our data also showed that CCNI2 depletion decreases cell proliferation. Depletion of CCNI has been shown to cause G2/M arrest and inhibit cell proliferation^23^. However, our data from two different cell lines suggest that although CCNI depletion affects cell cycle progression, it does not cause G2/M arrest or affect cell proliferation. The detailed molecular mechanisms by which CCNI and CCNI2 differently affect cell cycle remain elusive. One possibility is that CCNI and CCNI2 compete with each other for CDK5 binding and retain CDK5 in different subcellular compartments, where CDK5 affects cell cycle through phosphorylating different target proteins. Further investigation is needed to fully understand the mechanism how CCNI and CCNI2 differently regulate cell cycle.

## Materials and Methods

### DNA constructs and antibodies

All animal experiments were approved by the Ethics Committee of Shandong University and were performed in accordance with the relevant guidelines and regulations. The coding sequences of human CDK5 and CCNI, chicken CDK5, CCNI, and CCNI2 were amplified by PCR and cloned into expression vectors pEGFP-C2 or modified pEGFP-C2 (EGFP replaced with Myc or Flag). Human CCNI2 cDNA was obtained from Cyagen Biosciences Inc. Human CCNA1 cDNA was obtained from ViGene Biosciences Inc. Rabbit polyclonal anti-CDK5 antibody (Cat. No. sc-173, 1:1000 diluted) was from Santa Cruz, and rabbit polyclonal anti-CCNI2 antibody (Cat. No. 18822-1-AP, 1:1500 diluted) was from Proteintech. Rabbit monoclonal anti-phospho-CDK5 substrate antibody mix (Cat. No. 9477, 1:1000 diluted) was from Cell Signaling Technology. Rabbit polyclonal anti-ACTIN antibody (Cat. No. P30002, 1:5000 diluted) and mouse monoclonal anti-GFP antibody (Cat. No. M20004, 1:5000 diluted) were from Abmart. Mouse polyclonal anti-GAPDH antibody (Cat. No. MAB374, 1:5000 diluted) was from Millipore. Mouse monoclonal anti-Myc antibody (Cat. No. M4439, 1:5000 diluted) and mouse monoclonal anti-Flag antibody (Cat. No. F1804, 1:5000 diluted) were from Sigma-Aldrich.

### Yeast two-hybrid screen

The screen was performed as described previously^39^,^40^. Chicken CDK5 cDNA was cloned into vector pBD-GAL4 Cam (Stratagene) to express the bait protein. The yeast strain AH109 (Clontech) was sequentially transformed with the bait plasmid and a chicken cochlear cDNA library in the HybriZAP two-hybrid vector^41^. Totally 1.6 × 10^6^ transformants were selectively screened using *HIS3* as the reporter gene in the presence of 2.5 mM of 3-amino-1,2,4-triazole, and positive colonies were further tested for activation of two other reporter genes *ADE2* and *lacZ*. The pAD-GAL4-based phagemid vectors in triple-positive yeast colonies were recovered and cDNA sequence was determined by Sanger sequencing.

### Western blot

Dissected E18.5 chicken tissues or cultured cells were homogenized in ice-cold lysis buffer consisting of 150 mM NaCl, 50 mM Tris at pH 7.5, 1% (vol/vol) Triton X-100, 1 mM PMSF, and 1× protease inhibitor cocktail (Roche). After centrifugation at 4 °C, the supernatant was collected and separated by polyacrylamide gel electrophoresis (PAGE), then transferred to PVDF membrane and incubated with corresponding primary antibodies, followed by incubation with secondary antibodies (Bio-Rad). The signals were detected with the ECL system (Cell Signaling Technology).

### Cell transfection and co-immunoprecipitation (co-IP)

Cultured cells were transfected with expression vectors using jetPRIME Transfection Agent (Polyplus, Cat. No. PT-114-15) according to the manufacturer’s instructions, then washed with phosphate-buffered saline (PBS) 24 hours after transfection and lysed in ice-cold lysis buffer consisting of 150 mM NaCl, 50 mM Tris at pH 7.5, 1% (vol/vol) Triton X-100, 1 mM PMSF, and 1× protease inhibitor cocktail (Roche). After centrifugation at 4 °C, the supernatant was collected and incubated with immobilized anti-Myc antibody (Sigma-Aldrich, Cat. No. E6654) at 4 °C overnight. Immunoprecipitated proteins were washed with lysis buffer and separated by polyacrylamide gel electrophoresis (PAGE), then transferred to PVDF membrane and examined using western blot.

### Immunofluorescence

Transfected cells growing on Gelatin-coated glass cover slips were fixed with 4% paraformaldehyde (PFA) in PBS for 15 minutes, followed by 10 minutes PBS washes. For nuclei staining, cells were incubated with DAPI (Gen-View Scientific Inc.) for 1 hour, followed by three 10 minutes PBS washes, then mounted in Glycerol/PBS (1:1). The cells were imaged with a confocal microscope (LSM 700, Zeiss).

### Kinase assay

HEK293 cells were transfected with expression vectors and lysed in ice-cold lysis buffer consisting of 150 mM NaCl, 50 mM Tris at pH 7.5, 1% (vol/vol) Triton X-100, 1 mM PMSF, and 1× protease inhibitor cocktail (Roche). The supernatant was collected and incubated with immobilized anti-Flag antibody (Sigma-Aldrich, Cat. No. A2220) at 4 °C overnight, then washed four times with cold cell lysis buffer and subjected to CDK5 kinase assay using purified histone H1 protein (Millipore) as a substrate. The reaction mixture contains 20 mM MOPS (pH 7.2), 5 mM MgCl_2_, 500 μM histone H1 protein, 100 μM ATP, and 2.5 μCi γ-^32^ATP. The reaction continued for 30 min at room temperature and was stopped by the addition of 2× sample buffer, then separated by SDS-PAGE, followed by analysis with autoradiography. For PAK1 phosphorylation assay, immunoprecipitated Flag-PAK1 was separated by SDS-PAGE, followed by western blot analysis using anti-phospho-CDK5 substrate antibody (Cell Signaling Technology, Cat. No. 9477).

### Cell synchronization

For synchronization at late G1 phase, HeLa cells were plated at a density of 3 × 10^5^ cells per 60 mm dish and cultured for 24 hours. After exposure to 2 mM thymidine for 18 hours, cells were washed with PBS and incubated in fresh medium for 10 hours, then exposed to 2 mM thymidine again for 12 hours. To release cells from the late G1 arrest, cells were washed with PBS and incubated in fresh medium for different periods.

### Reverse transcription-polymerase chain reaction (RT-PCR)

Total RNA of HeLa cells were extracted using RNeasy Micro Kits (Qiagen) according to the manufacturer’s protocol. Reverse transcription (RT) was carried out at 42 °C for 1 hour in a 20 μl reaction mixture containing 1 μg of total RNA, 10 pmol of oligo-dT, and 200 units of Super-Script III reverse transcriptase (Invitrogen). Polymerase chain reaction (PCR) was performed using the cDNA as template with the following primers: *CDK5* forward primer, CGCCGCGATGCAGAAATACGAGAA, reverse primer, TGGCCCCAAAGAGGACATC (439 bp); *CCNI* forward primer, AAGGTATTGGCAAGAGACAGTTTC, reverse primer, GGTTGCAGGCCATACAGTGA (244 bp); *CCNI2* forward primer, CTGGACAGACTGCACTGGGAC, reverse primer, CTCCAGCTCTAAGGTGATGAT (233 bp); *CCNE* forward primer, GACCGGTATATGGCGACACAAGAA, reverse primer, GTCGCACCACTGATACCCTGAAAC (449 bp); *CCNB* forward primer, GCCCCTGCAGAAGAAGACC, reverse primer, GTAGAGGCCGACCCAGACC (498 bp); *β*-*actin* forward primer, CTCCATCCTGGCCTCGCTGT, reverse primer, GCTGTCACCTTCACCGTTCC (268 bp). To obtain the optimal sensitivity and specificity, cycle lengths for different PCR reaction sets were adjusted between 23 and 38 cycles, and annealing temperatures were adjusted between 56 and 64 °C. The PCR products were separated by electrophoresis on agarose gel.

### Quantitative real-time PCR

Quantitative real-time PCR was carried out using SYBR^®^ Premix Ex Taq^TM^ system (Perfect Real Time, Takara). The primers and template were the same as in RT-PCR. Amplification and detection were run in a Sequence Detection System SLA-3296 (Bio-Rad) with an initial cycle of 95 °C for 10 s followed by 40 cycles of 95 °C for 5 s, 64 °C for 30 s and 72 °C for 20 s. All PCR reactions were performed in triplicate. Negative control samples (without template) were processed in the same way. The specificity of the amplifications was verified by melting curve analysis. Relative quantization of ionic channel gene expression normalized to β-actin was calculated according to the 2^−ΔΔCT^ method.

### RNA interference and flow cytometry

CCNI and CCNI2 siRNAs were obtained from Sigma-Aldrich (si-ccni-1: SASI_Hs01_00052221; si-ccni-2: SASI_Hs01_00052222; si-ccni2-1: SASI_Hs02_00308672; si-ccni2-2: SASI_Hs01_00030733). Sequence of CDK5 siRNAs are as follow: UAUGACAGAAUCCCAGCCCTT, GGGCUGGGAUUCUGUCAUATT^42^. For efficient gene silencing, siRNAs were transfected twice. Briefly, HeLa or A549 cells were transfected with 30 nM siRNAs using jetPRIME^®^ Transfection Agent (Polyplus) according to the manufacturer’s instruction. After culture for 24 hours, the second transfection was performed. Cells were cultured for another 24 hours, and then collected and fixed with 70% ethanol at −20 °C overnight. They were incubated with 0.5 ml of PBS buffer containing 5 μg PI (Sigma-Aldrich) and 50 μg RNase (Dingguo), and the DNA content was measured using Amnis ImageStreamX Mark II Flow Cytometer (Merck Millipore). For each sample, 1.5 × 10^4^ events were analyzed and the data were plotted using ModFit software (Verity Software House).

### Bromodeoxyuridine (BrdU) incorporation assay

HeLa or A549 cells were transfected with siRNA as described above. Seventy-two hours after transfection, cells were treated with 10 μM BrdU (Sigma-Aldrich) for 2 hours. Cells were then fixed with 4% paraformaldehyde, stained with mouse monoclonal anti-BrdU antibody (Sigma-Aldrich) and DAPI (Gen-View Scientific Inc.). BrdU-positive nuclei were examined using a florescent microscope (IX53, Olympus).

### Cell proliferation assay

HeLa or A549 cells (1 × l0^4^ cells/well) transfected with siRNA were seeded in 96-well plates. At different time points (24, 48, 72, and 96 hours) after transfection, cells in certain wells were incubated with 0.5 mg/ml MTT reagent (Gen-View Scientific Inc.) at 37 °C for 4 hours. Then the supernatant was removed, and the converted dye was solubilized with 150 μl DMSO per well. Absorbance at 570 nm was measured using a micro-plate reader (MULTISKAN-FC, Thermo Scientific). The cell proliferation/viability was indicated by the obtained numerical values using the following equation: cell viability = [optical density (OD) value of test group − OD value of blank group]/(OD value of control group − OD value of blank group). The experiment was performed in triplicate.

### Statistical analysis

Data were shown as mean ± SD from at least 3 independently performed experiments. Student’s t test was used for analysis. P < 0.05 was considered statistically significant.

## Additional Information

**How to cite this article**: Liu, C. *et al*. Cyclin I-like (CCNI2) is a cyclin-dependent kinase 5 (CDK5) activator and is involved in cell cycle regulation. *Sci. Rep.* **7**, 40979; doi: 10.1038/srep40979 (2017).

**Publisher's note:** Springer Nature remains neutral with regard to jurisdictional claims in published maps and institutional affiliations.

## Supplementary Material

Supplemental Materials

## Figures and Tables

**Table 1:** Potential CDK5-binding partners identified from yeast two-hybrid screening.

GenBank accession No.	Prey protein	Prey redundancy
XP_42059	Cyclin I (CCNI)	3
XP_001234830	Cyclin I-like (CCNI2)	2

CDK5 was used as bait to screen a chick cochlear cDNA library. Totally five positive clones were obtained, encoding CCNI and CCNI2.
