# Erratum: Cyclin I-like (CCNI2) is a cyclin-dependent kinase 5 (CDK5) activator and is involved in cell cycle regulation

**DOI:** 10.1038/srep44164

**Published:** 2017-03-15

**Authors:** Chengcheng Liu, Xiaoyan Zhai, Bin Zhao, Yanfei Wang, Zhigang Xu

Scientific Reports 7: Article number: 4097910.1038/srep40979; published online: 01 23 2017; updated: 03 15 2017

This Article contains errors in the Reference list. Reference 33 is incorrectly listed as reference 26 and references 26–32 are incorrectly listed as references 27–33 respectively. The correct references are listed below:

26 Zhang, J. *et al*. Nuclear localization of Cdk5 is a key determinant in the postmitotic state of neurons. *Proc. Natl. Acad. Sci. USA* **105**, 8772–8777 (2008).

27 Liang, Z. *et al*. Cdk5 regulates activity-dependent gene expression and dendrite development. *J. Neurosci*. **35**, 15127–15134 (2015).

28 Ino, H. & Chiba, T. Intracellular localization of cyclin-dependent kinase 5 (CDK5) in mouse neuron: CDK5 is located in both nucleus and cytoplasm. *Brain Res*. **732**, 179–185 (1996).

29 Asada, A. *et al*. Myristoylation of p39 and p35 is a determinant of cytoplasmic or nuclear localization of active cyclin-dependent kinase 5 complexes. *J. Neurochem*. **106**, 1325–1336 (2008).

30 Asada, A., Saito, T. & Hisanaga, S. Phosphorylation of p35 and p39 by Cdk5 determines the subcellular location of the holokinase in a phosphorylation-site-specific manner. *J. Cell Sci*. **125**, 421–3429 (2012).

31 Saito, T. *et al*. p25/cyclin-dependent kinase 5 promotes the progression of cell death in nucleus of endoplasmic reticulum-stressed neurons. *J. Neurochem*. **102**, 133–140 (2007).

32 Hagmann, H. *et al*. Cyclin I and p35 determine the subcellular distribution of Cdk5. *Am. J. Physiol. Cell Physiol*. **308**, C339–347 (2015).

33 Xue, Y. *et al*. GPS 2.1: enhanced prediction of kinase-specific phosphorylation sites with an algorithm of motif length selection. *Protein Eng. Des. Sel*. **24**, 255–260 (2011).

In the Discussion section under “CCNI2 regulates cell cycle”, the references following the sentence “Several groups showed independently that CDK5 could regulate cell cycle^26,27,34–37^”

Should read:

“Several groups showed independently that CDK5 could regulate cell cycle^26,34–37^”.

