# Supplementary material for: Cyclin I-like (CCNI2) is a cyclin-dependent kinase 5 (CDK5) activator and is involved in cell cycle regulation
Source: Sci Rep. 2017 Jan 23;7:40979. doi: 10.1038/srep40979 (PMC5256034; doi:10.1038/srep40979)
Supplement: Supplemental Materials [file srep40979-s1.pdf]

Cyclin I-like (CCNI2) is a cyclin-dependent kinase 5 (CDK5) activator and is involved in cell cycle regulation

Chengcheng Liu[1], Xiaoyan Zhai[1], Bin Zhao[1], Yanfei Wang[1], Zhigang Xu[1,*]

[1]Shandong Provincial Key Laboratory of Animal Cells and Developmental Biology, Shandong University School of Life Sciences, Jinan, Shandong 250100, China

[*]Author for correspondence (xuzg@sdu.edu.cn)

Keywords: CCNI2, CDK5, CCNI, cell cycle, kinase

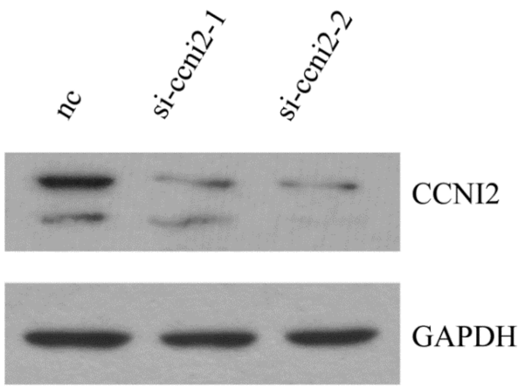

Supplemental figure S1. CCNI2 knockdown by siRNAs. Cell lysates of siRNA-transfected HeLa cells were subjected to western blot using a polyclonal anti-CCNI2 antibody. GAPDH was used as control.

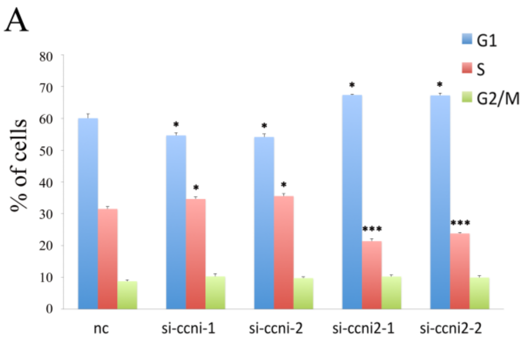

	nc	si-ccni-1	si-ccni-2	si-ccni2-1	si-ccni2-2
G1	60.0±1.7	54.6±1.7	54.1±2.2	67.4±0.3	67.2 ±0.7
S	31.5±1.0	34.6 ± 1.0	35.6±1.1	21.4±1.6	23.8±0.3
G2/M	8.8±0.6	10.3±1.2	9.8±0.5	10.3±0.7	10.0 ±0.6

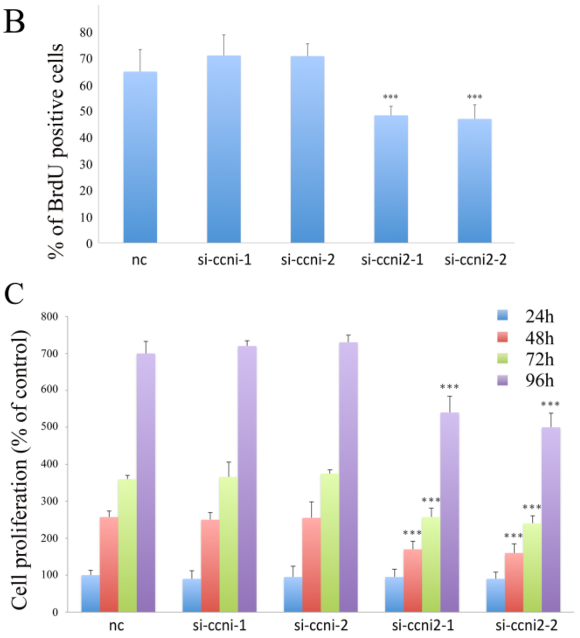

Supplemental figure S2. Knockdown of CCNI2 decreases cell cycle progression and cell proliferation of A549 cells. (A) Cell cycle profiles of CCNI-depleted and CCNI2-depleted A549 cells. (B) BrdU incorporation of CCNI-depleted and CCNI2-depleted A549 cells. (C) Cell proliferation of CCNI-depleted and CCNI2-depleted A549 cells was examined by MTT assay. The bar graphs and the table show quantification of the results, with each value represents the mean ± SD of three independent experiments. Statistical significance is shown using the Student *t* test analysis; *$P < 0.05$; **$P < 0.01$; ***$P < 0.001$.

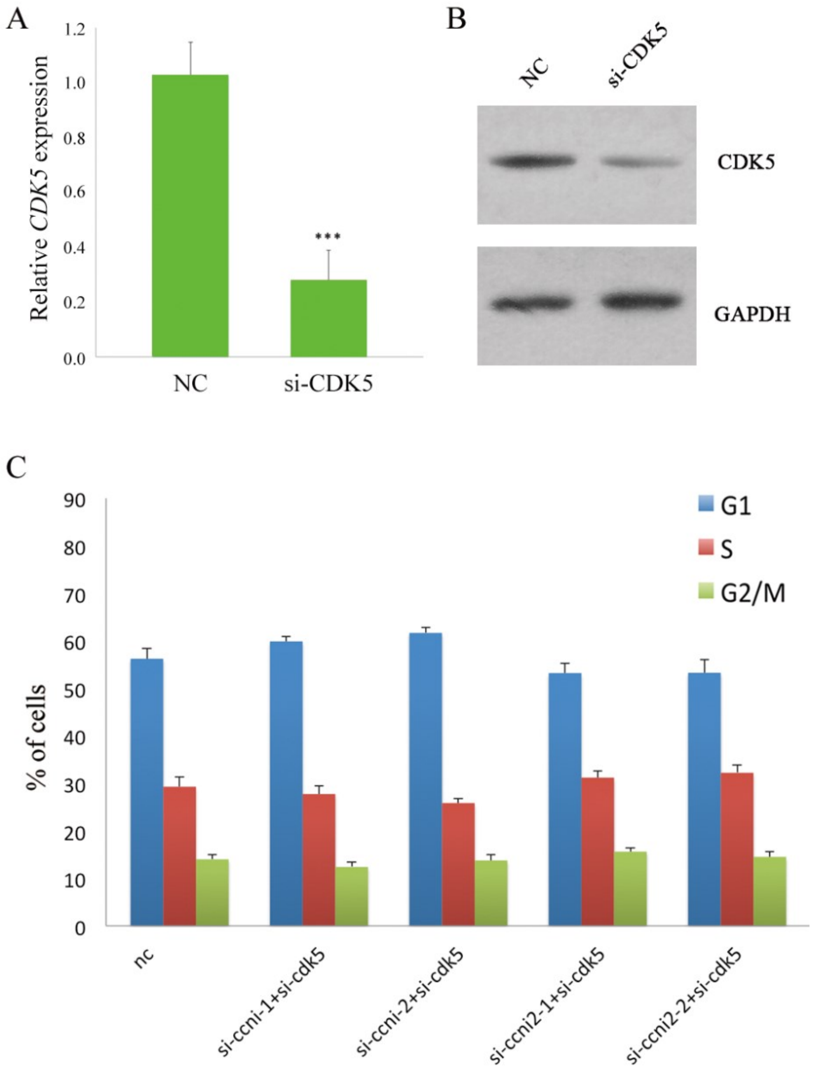

Supplemental figure S3. The effect of CCNI/CCNI2 depletion on cell cycle is dependent on CDK5. The efficiency of siRNA against CDK5 was examined by quantitative PCR (A) and western blot (B), respectively. (C) Cell cycle profiles of CCNI/CDK5-depleted and CCNI2/CDK5-depleted HeLa cells. When CDK5 siRNA is present, CCNI or CCNI2 depletion does not affect cell cycle progression. The bar graph shows quantification of the results, with each value represents the mean ± SD of three independent experiments.

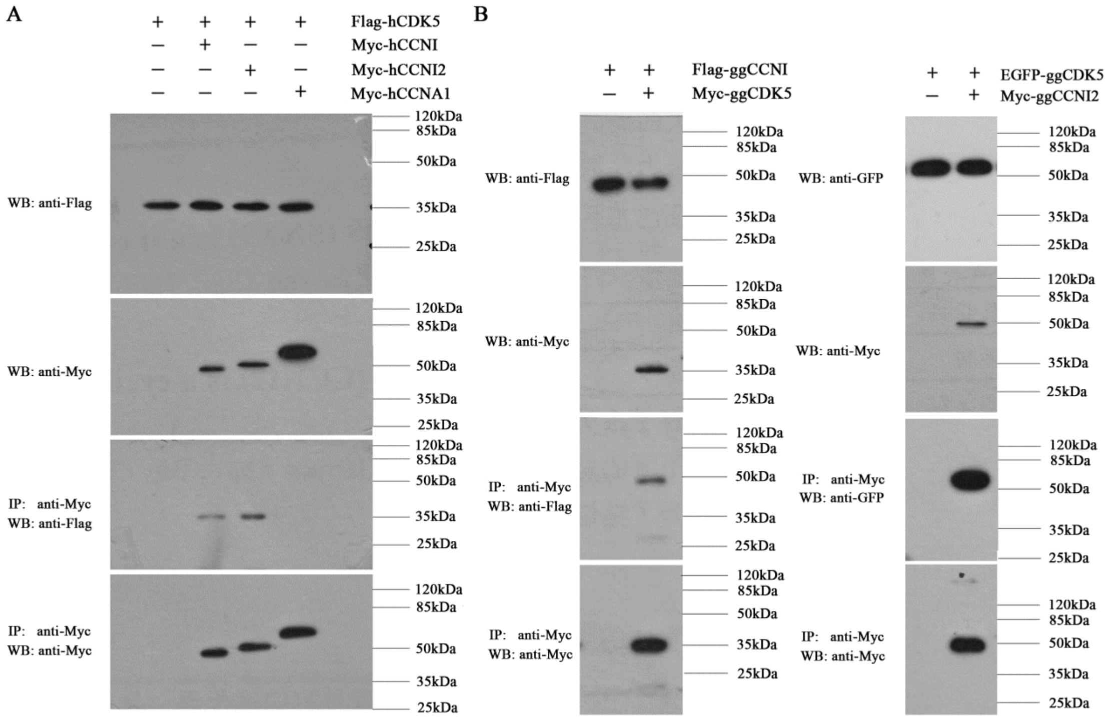

Supplemental figure S4. Uncropped blots corresponding to figure 2.

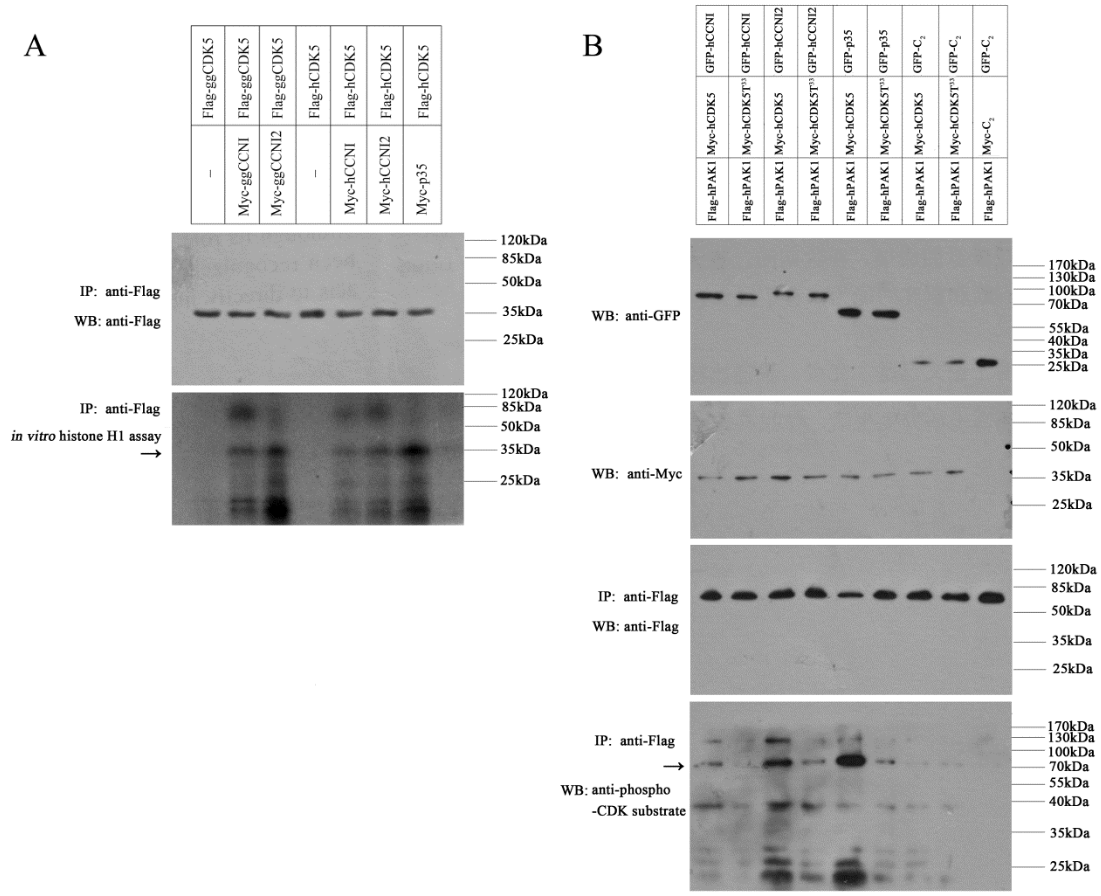

Supplemental figure S5. Uncropped blots corresponding to figure 4.
